# Design and optimisation of magnetically-tunable hybrid piezoelectric-triboelectric energy harvester

**DOI:** 10.1038/s41598-021-83776-y

**Published:** 2021-02-24

**Authors:** Satish Rao Ganapathy, Hanim Salleh, Mohammad Khairul Azwan Azhar

**Affiliations:** Institute of Sustainable Energy, Universiti Tenaga Nasional (UNITEN), Jalan IKRAM-UNITEN, 43000 Bangi, Selangor Malaysia

**Keywords:** Engineering, Sensors, Energy harvesting, Devices for energy harvesting, Renewable energy

## Abstract

The demand for energy harvesting technologies has been increasing over the years that can be attributed to its significance to low power applications. One of the key problems associated with the available vibration-based harvester is the maximum peak power can only be achieved when the device frequency matches the source frequency to generate low usable power. Therefore, in this study, a magnetically-tunable hybrid piezoelectric-triboelectric energy harvester (MT-HPTEH) was designed and optimised. Four key design factors: mass placement, triboelectric surface area, extension length and magnetic stiffness were investigated and optimised. The voltage generation from piezoelectric and triboelectric mechanisms was determined individually to understand the effect of each design factor on the mechanisms. An output power of 659 µW at 180 kΩ at 44 Hz was obtained from the optimised MT-HPTEH with a theoretical–experimental discrepancy of less than 10%. The added magnetically-tunable feature enabled the harvester to work at the desired frequency range with an open circuit voltage between 7.800 and 20.314 V and a frequency range from 38 to 54 Hz. This MT-HPTEH can power at least six wireless sensor networks and can be used for low power applications such as RFID tags. Future work may include designing of energy-saving and sustainable harvester.

## Introduction

The rapid growth of the electronic industry is evident with increasing demand for portable electronic devices such as mobile phones, gadgets, and tablets which are convenient for users^1^. Conventionally, mobile devices operate using electrochemical power sources, for instance, the commonly used lithium batteries. However, the conventional electrochemical batteries require regular recharging and replacement which significantly affects the operation lifetime and energy-sustainability of the device. To resolve this issue, various studies have been carried out for the integration of built-in, nano-sized energy generator into the mobile devices^2^. The development of mobile devices equipped with mobile energy harvester which has self-charging capability can resolve numerous challenges of powering complex devices in recent years^3^. The self-powering capability can be achieved via harvesting ambient environmental energy readily available in the natural ecosystem by converting mainly mechanical energy to electricity^4^.

The invention of triboelectric nanogenerator (TENG) has attracted researchers in the energy-harvesting field^5–7^. Its outstanding efficiency, reliability and cost-effectiveness^8^ has promoted TENG application in mobile devices^9^, light-emitting diodes (LEDs)^10^, motion sensors^11^, vacuum electronic devices^12^, and shoe-based miniaturised nanogenerators^13,14^. To further enhance the energy-harvesting performance of TENG, hybrid energy harvesters were integrated with other energy harvesters such as electromagnetic^9,15^, piezoelectric^16^, solar^17^, and electrochemical cells^18^. Amongst the reported hybridisations, piezoelectric and triboelectric harvesters were of growing research interest as the mechanical conversion of energy can be achieved flexibly using both piezoelectric and triboelectric mechanisms^19–21^. Besides, the performance of hybridised energy harvester can be further enhanced by incorporating magnetic force in the energy harvesting mechanism to increase the frequency of triboelectric occurrence during energy-harvesting cycles^11,22,23^.

In this study, a magnetically-tunable hybrid piezoelectric-triboelectric energy harvester (MT-HPTEH) was designed and optimised. The key figure-of-merit for the design is the output voltage of MT-HPTEH, where a maximum output voltage should be achieved under the resonance vibration of the MT-HPTEH. Various factors that can influence the total output voltage of MT-HPTEH were investigated and optimised. Besides, both computational and experimental outcomes were evaluated and compared to ensure the validity of the obtained output voltages.

## Theoretical background

Theoretical computation is often used to benchmark against the experimental outcome to verify the accuracy of measured output voltage of MT-HPTEH. In this study, piezoelectric and triboelectric energy harvesting mechanisms were used and the theoretical computation of both mechanisms was as shown in Eqs. (1) to (6). The main figure-of-merit of the designed MT-HPTEH was to estimate the highest possible output open-circuit voltage achieved by the prototype.

For the piezoelectric mechanism, the open-circuit voltage was expressed as^24,25^:

1 $${V}_{oc}=\frac{3{d}_{33}L}{2{\varepsilon }_{33}wt\left(1-\frac{{k}_{33}}{4}\right)}F$$

where, ${d}_{33}$ is the piezoelectric constant, ${k}_{33}$ is the coupling coefficient, ${\varepsilon }_{33}$ is the dielectric constant of piezoelectric material, $t$ is the thickness of the beam, $w$ is the width of the beam, and $F$ is the mechanical force acting on the beam. The maximum power output of the piezoelectric mechanism was expressed as^26^:

2 $${P}_{{e}_{max}}=\frac{m\mathrm{aQ }}{4{\omega }_{n}}$$

where *m* is the mass on the beam, $\mathrm{a}$ is the acceleration, *ω*_*n*_ is the natural frequency of the system and *Q* is the quality factor. Meanwhile, the triboelectric mechanism involves induction of electron transfer upon physical friction between two different surfaces. The open-circuit voltage of triboelectric mechanism was expressed as^27^:

3 $${V}_{oc}=\frac{{\sigma}x(t)}{{\varepsilon }_{0}}$$

where *σ* is the surface charge density, *x* is the gap separation between two surfaces at a given time, *t*, and ${\varepsilon }_{0}$ is the dielectric constant of the triboelectric material. Using the triboelectric surface charge density, optimum load resistance, ${R}_{opt}$, output current, ${I}_{SC}$ and power output, *P*, were calculated as follows^28^:

4 $${R}_{opt}=\frac{{\left({d}_{0}+{x}_{max}\right)}^{2}}{{{{\mathrm{S}}v\varepsilon }}_{0}}$$

5 $${I}_{SC}=\frac{{{S\sigma }{d}_{0}{v}({t})}_{.}}{{\left({d}_{0}+x(t)\right)}^{2}}$$

6 $$P={{({I}_{SC})}^{2}{R}_{opt}}_{.}$$

where *S* is the surface area of triboelectric layers, *d*_*0*_ is the effective thickness of materials, *x*_*max*_ is the maximum separation gap between materials, and *v* is the velocity of the contact separation process.

## Experimental methods

**Figure 1:**
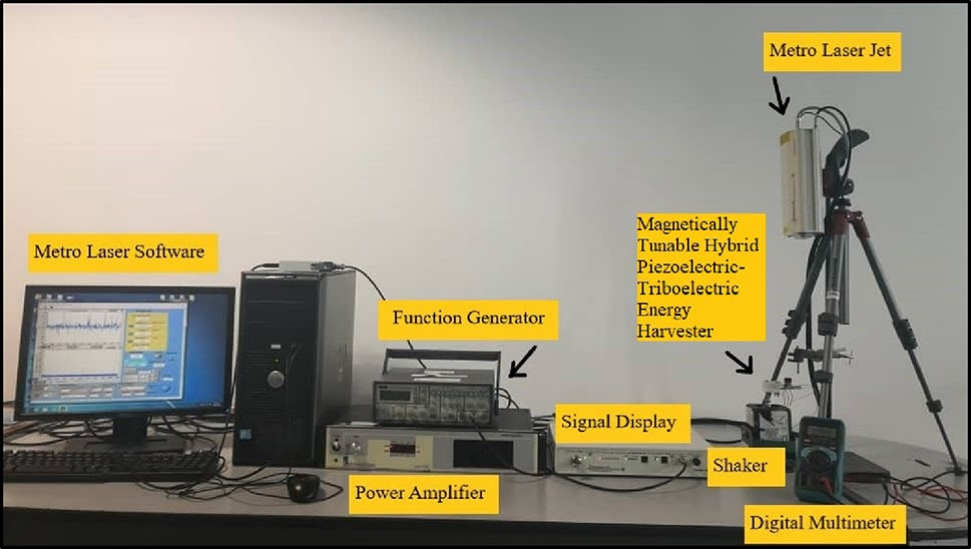
Experimental setup for the output voltage measurement of MT-HPTEH.

Figure 1 shows the output voltage measurement setup of magnetically-tunable hybrid piezoelectric-triboelectric energy harvester (MT-HPTEH). A MT-HPTEH prototype was first placed on a shaker and positioned towards a metro laser jet. The shaker was then triggered by adjusting the output from the function generator. The function generator was used to adjust the vibration frequency and amplitude of the shaker where the parameters can be specified using a metro laser software. The metro laser jet projected laser onto the harvester for vibration frequency detection. When the frequency of shaker has been stabilised, a digital multi meter was used to measure the voltage produced from the corresponding vibration frequency of the shaker. The characterisation steps were repeated for various sets of variables.

**Figure 2:**
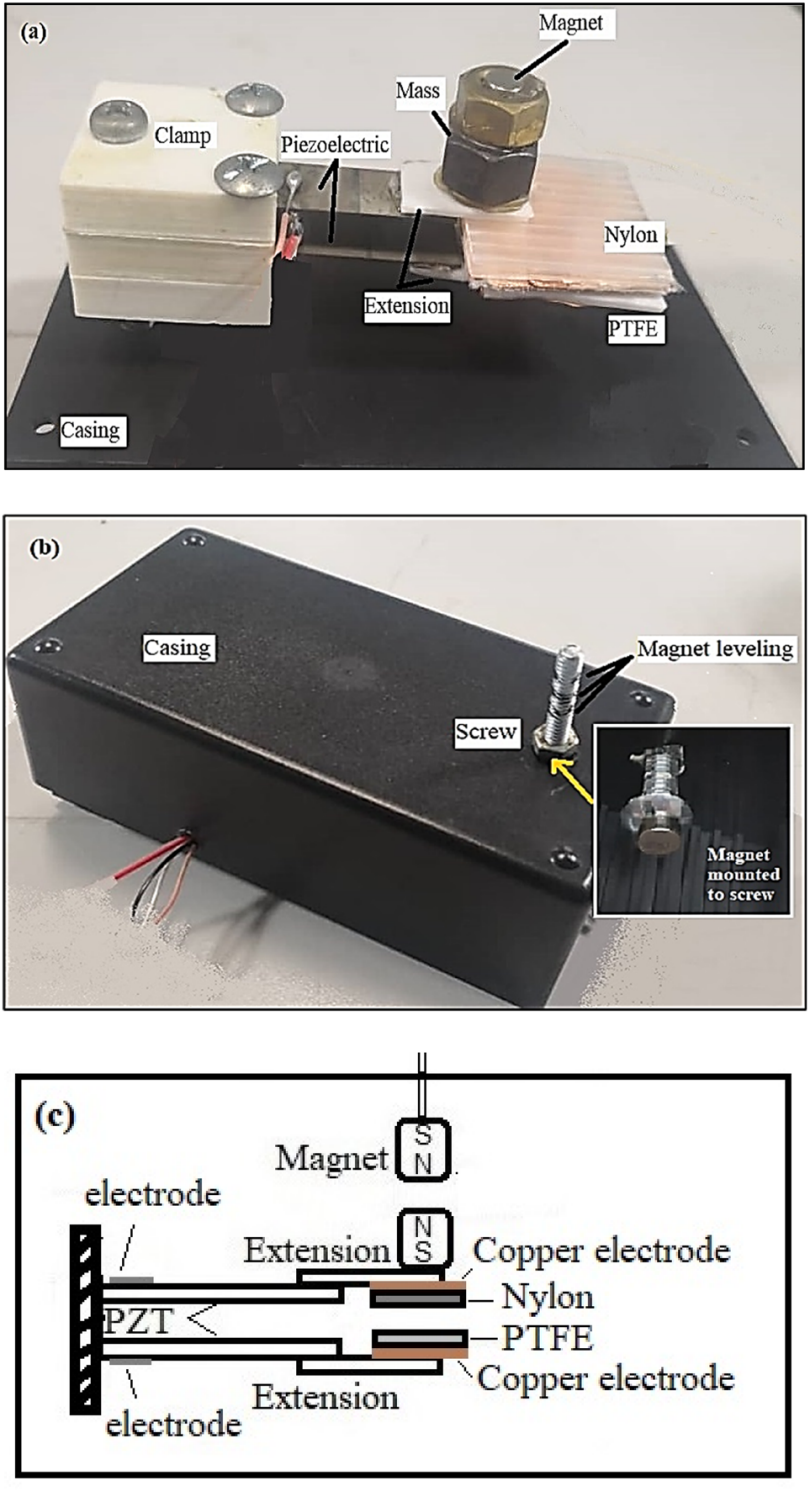
(**a**) Interior image (**b**) exterior image and (**c**) schematic diagram of MT-HPTEH prototype.

A MT-HPTEH prototype developed is as illustrated in Fig. 2. A PSI-5H4E piezoelectric material with lead Zirconate Titanate structure was used as a piezoelectric beam. Upon exertion of mechanical stress by the shaker, two piezoelectric beams produced electrical energy which was measured as an open-circuit voltage (dimension: 3 × 3 × 0.025 cm; dielectric constant: 3800 V/m; coupling efficiency: 0.75). Polytetrafluoroethylene (PTFE) sheet was used as the triboelectric material due to its low mechanical friction, high melting point, and high chemical stability. Different masses were placed on the beams to alter the vibration frequency of the MT-HPTEH. Then, a pair of magnets were placed on the top beam of the MT-HPTEH prototype to tune the frequency at peak output voltage via a magnetic repulsive force.

The design of the MT-HPTEH prototype was optimised considering the following key factors: mass placed on the extension beams, the surface area of the PTFE triboelectric layers, extension length, and the magnetic stiffness. The magnetic stiffness can be defined as the gap between two magnets integrated on the upper beam induced by repulsive magnetic forces, as shown in Fig. 2b. The parameter investigations were carried out by comparing the theoretical computation and experimental measurement outcomes of the open-circuit voltage induced by top piezoelectric beam, bottom piezoelectric beam, and triboelectric mechanism. The total voltage of the MT-HPTEH was then calculated from the sum of measured voltage values of each mechanism.

## Results and discussion

The key design factors that were used to design and optimise MT-HPTEH were investigated based on two different energy harvesting mechanisms: piezoelectric and triboelectric. The effect of mass placement on the piezoelectric beams, the triboelectric surface area of the MT-HPTEH, nylon extension length and magnetic stiffness of the magnetic-assistance in MT-HPTEH on the energy-harvesting performance of MT-HPTEH were discussed in “Effect of mass” to “Effect of Magnetic Stiffness” sections. The power generation performance of MT-HPTEH was summarised in “Overall Performance of Optimised MT-HPTEH” section. .

### Effect of mass

To investigate the effect of mass placement on the energy-harvesting performance of MT-HPTEH, the natural frequency of the energy harvester was determined for different mass placement. As the primary working mechanism of piezoelectric involves deflection of beam body to generate electrical power, the mechanical vibration and natural frequency of the MT-HPTEH play a vital role in the generation of electrical output. To establish the theoretical computation considering mass placement as the key design factor, the piezoelectric beam stiffness can be expressed as^29^:

7 $${K}_{pzt}=\frac{3EI}{{({L}_{pzt})}^{3}}=\frac{3Ew{d}^{3}}{{12({L}_{pzt})}^{3}}$$

where *E* is Young’s modulus of the piezoelectric beam, *I* is the moment of inertia, *L* is the length of the beam, *w* is the width of the beam and *d* is the thickness of the beam.

**Figure 3:**
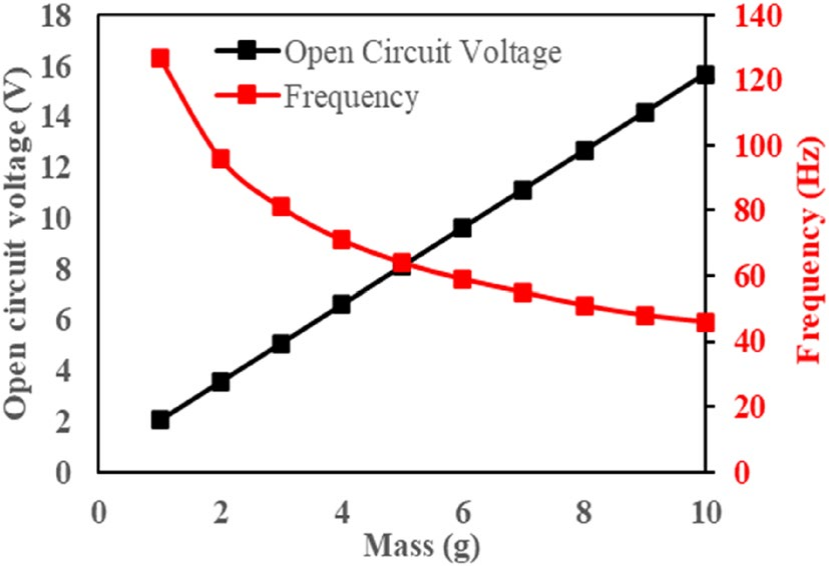
Effect of mass on natural frequency and open-circuit voltage of piezoelectric beam (computation outcomes).

In the case of MT-HPTEH, it was fine-tuned by an external magnetic force as illustrated in Fig. 2b. As a result, both ${K}_{pzt}$ and magnetic stiffness shall be taken into account for the calculation of natural frequency. The magnetic stiffness of the MT-HPTEH can be expressed as^30^:

8 $${K}_{mag}=\left|\frac{{F}_{mag}}{d}\right|=\left|\left[\frac{{{B}_{r}}^{2}{{A}_{m}}^{2} {({L}_{mag}+{r})}^{2}}{2{\pi d}{\mu }_{0}{{L}_{mag}}^{2}}\right]\left[\frac{1}{{d}^{2}}+\frac{1}{{({d+2L}_{mag})}^{2}}-\frac{2}{{({d+L}_{mag})}^{2}}\right]\right|$$

where *F*_*mag*_ is the magnetic force, *B*_*r*_ is the flux density between the magnets, *A*_*m*_ is the surface area of the magnet, *r* is the radius, *μ*_*0*_ is the permeability of the medium between the magnets, *L*_*mag*_ is the length of the magnet, d is the distance separating both magnets end to end. By using the calculated ${K}_{pzt}$ and ${K}_{mag}$ values the natural frequency of the MT-HPTEH can be expressed as^31^:

9 $${\upomega }_{n}=\sqrt{\frac{{K}_{mag}+{K}_{pzt}}{{m}_{eff}}}$$

where the effective mass (*m*_*eff*_) refers to the total mass of the piezoelectric beam and triboelectric layers. By using Eqs. (7) to (9), the effect of mass on the natural frequency of the piezoelectric beam was calculated and the trend was exhibited in Fig. 3.

From Fig. 3, it can be observed that the natural frequency of the MT-HPTEH reduced with higher mass placed on the piezoelectric beam as computed using Eq. (9). Meanwhile, the calculated open-circuit voltage increased with increasing piezoelectric beam mass. As described earlier, the piezoelectric energy-harvesting mechanism involves mechanical deflection of the piezoelectric beam to generate electrical energy. As calculated using Eq. (1), higher mass resulted in higher deflection of the beam, generating higher open-circuit voltage.

**Figure 4:**
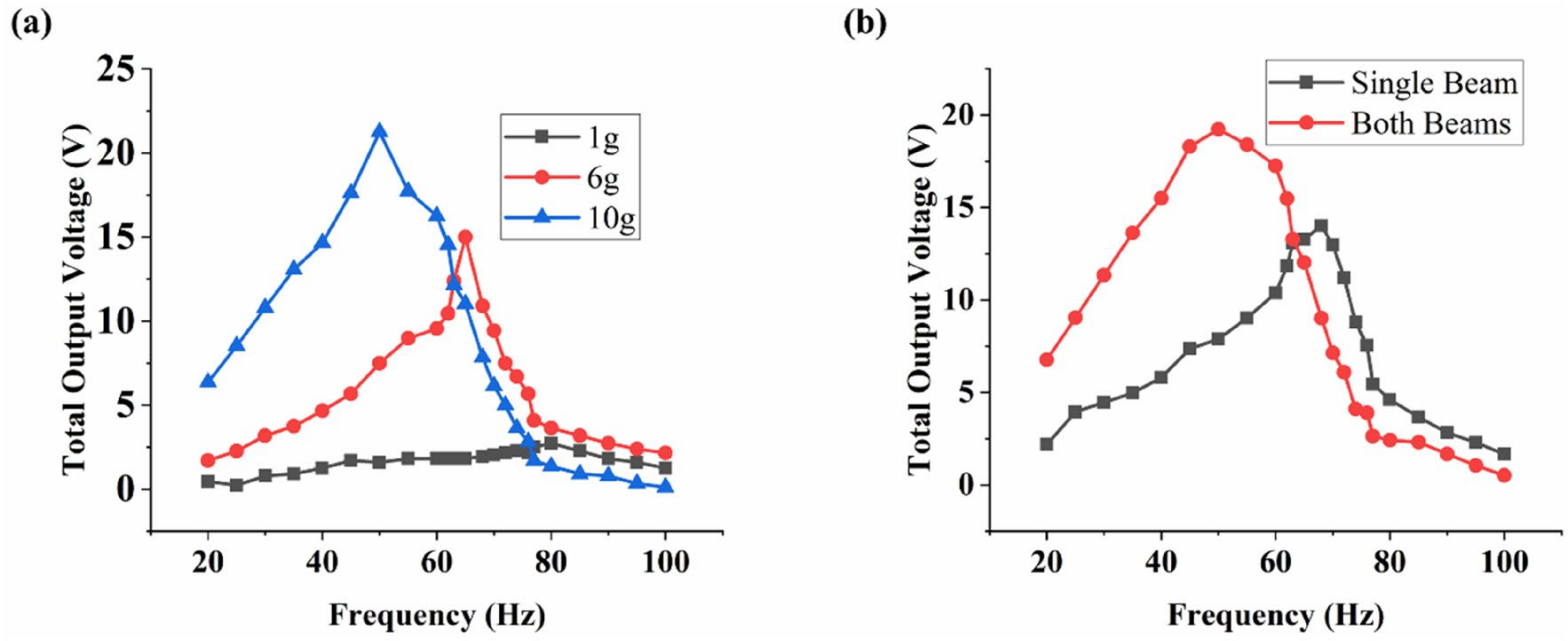
Total output voltage at (**a**) different masses (both beams), (**b**) different placement (10 g mass).

To understand better the mass placement effect, three data points (1, 6 and 10 g) from Fig. 3 were selected for experimental measurement of total output voltage using the MT-HPTEH prototype as shown in Fig. 4a.

From Fig. 4a, it can be noted that higher mass results in higher output voltage. A 10 g of mass induced the highest output voltage of 22 V, followed by 15 V and 2.5 V as affected by 6 g and 1 g masses, respectively. The natural frequency of MT-HPTEH reduced with increasing mass placement, where the low natural frequency of 45 Hz was obtained for 10 g mass placement. This finding was consistent with the computational outcomes as calculated using Eqs. (1) and (9), where the natural frequency reduced while output voltage increased with increasing mass (Fig. 3).

To further explore the possible variations in the MT-HPTEH design, the effect of mass placement on single piezoelectric beam (top beam only) and both piezoelectric beams (top and bottom beams) was analysed. A standard mass of 10 g was used as it produced the largest output voltage as shown in Fig. 4a. A comparison between total output voltages resulted as influenced by masses placed on single beam and both beams are as shown in Fig. 4b. From Fig. 4b, it can be observed that mass placement on both beams led to a higher output voltage of 20 V compared to the single beam. This can be attributed to the additional piezoelectric energy-harvesting mechanism from the bottom beam as described in Eq. (1). Nevertheless, apart from mass placement, improvement of triboelectric material surface area and extension addition can increase the output voltage.

The open-circuit voltage recorded as a result of the top piezoelectric beam, bottom piezoelectric beam, and triboelectric mechanisms were benchmarked against theoretical values. The theoretical and experimental open-circuit voltage values are compiled in Table 1. Based on values presented in Table 1, it can be noted that the open-circuit voltage from piezoelectric mechanism increased significantly with a higher mass as compared to voltage increment from the triboelectric mechanism. This observation is consistent with the theoretical outlines as indicated by Eqs. (1) and (2), where an increase in the mechanical force on cantilever beams in MT-HPTEH significantly affected the piezoelectric mechanism than the triboelectric mechanism. The percentage difference between the theoretical and the experimental output voltages ranged from 5 to 18%. The discrepancy between theoretical and experimental values can be ascribed to the empirical determination of triboelectric charge density and presence of external mechanical noise during the experiment^32,33^. However, the theoretical–experimental percentage differences were less than 20%, which suggest that the measured total output voltage from MT-HPTEH aligns well with the theoretical computation from Eq. (1).

**Table 1:** Theoretical and experimental voltages from the top piezoelectric beam, bottom piezoelectric beam, and triboelectric mechanism for different mass placements.

Mass (g)	Top piezoelectric beam open-circuit voltage (V)		Percentage difference (%)
	Theoretical	Experimental	
1.0	2.054	1.723	16.11
6.0	9.609	8.552	11.00
10.0	15.109	12.489	17.34

Mass (g)	Bottom piezoelectric beam open-circuit voltage (V)		Percentage difference (%)
	Theoretical	Experimental	
1.0	2.054	1.678	18.31
6.0	9.609	8.845	7.95
10.0	15.109	12.493	17.31

Mass (g)	Triboelectric mechanism open-circuit voltage (V)		Percentage difference (%)
	Theoretical	Experimental	
1.0	0.90	0.91	1.10
6.0	1.00	1.05	5.00
10.0	1.30	1.21	6.90

**Figure 5:**
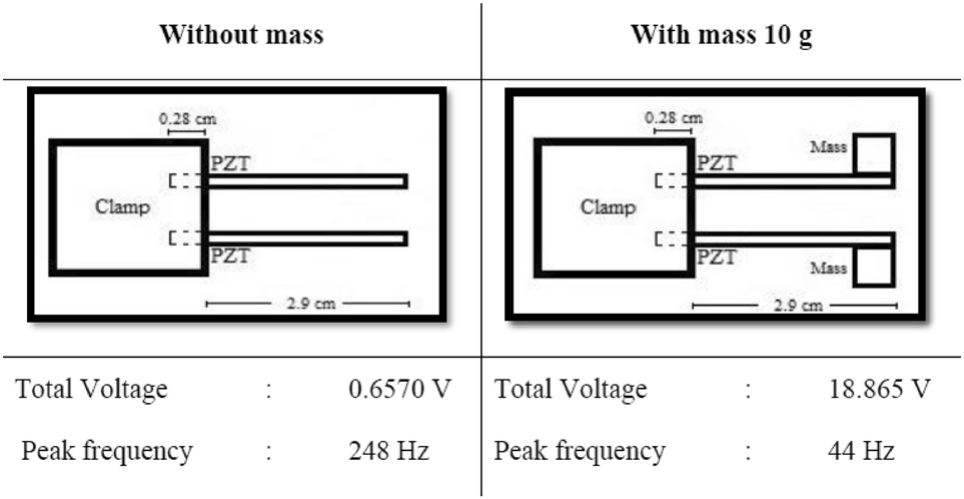
Performance enhancement of MT-HPTEH with optimised mass placement.

From Fig. 5, it can be observed that the output voltage of MT-HPTEH prototype increased significantly from 0.657 V to 18.865 V upon integration with 10 g of mass whereas the peak frequency reduced from 248 to 44 Hz, in line with the computation data (Fig. 3). This improvement can be attributed to the additional piezoelectric effect on the MT-HPTEH with output voltage from the triboelectric mechanism with the addition of 10 g of mass that affected the overall performance of the harvester as shown in Table 1.

### Effect of surface area

Besides mass placement, the surface area of the triboelectric layer of MT-HPTEH can be adjusted to optimise the output voltage of the energy harvester. As indicated in Eq. (3), the open-circuit voltage of MT-HPTEH correlates with its surface charge density, which can be directly related to the surface area of the triboelectric layer. In this study, Nylon and PTFE surface areas of 3, 4, and 9 cm^2^ were selected to investigate the effect of triboelectric layer surface area on the output voltage of MT-HPTEH.

**Figure 6:**
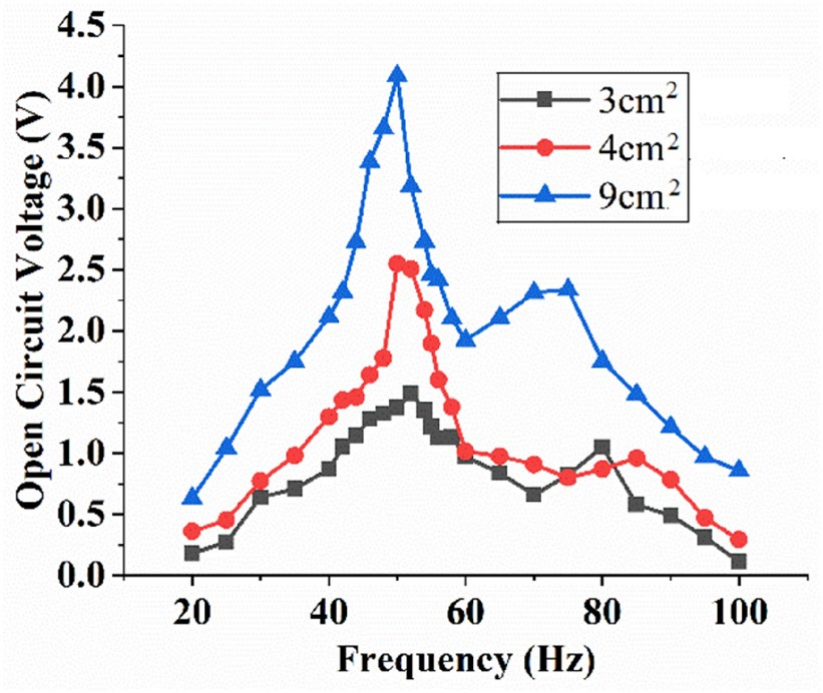
Effect of surface area on the triboelectric open-circuit voltage of MT-HPTEH.

Figure 6 shows the output voltage of MT-HPTEH using various Nylon and PTFE surface areas. The highest peak voltage was obtained at 4.134 V at a resonant frequency of 50 Hz for a 9 cm^2^ PTFE surface area. The obtained voltage was higher than the 3 cm^2^ and 4 cm^2^ counterparts that harvested 1.479 V at 52 Hz and 2.542 V at 50 Hz, respectively. The triboelectric surface area was noted to exert minimal influence on the natural frequency of MT-HPTEH, where the peak voltages occur at ~ 50 Hz as the surface area increases from 3 to 9 cm^2^. This indicates that change in triboelectric surface area imparts a relatively insignificant influence on the piezoelectric energy-harvesting mechanism as compared to the triboelectric mechanism. This finding is line with the theoretical background as shown in Eqs. (1) to (3) where the piezoelectric and triboelectric energy-harvesting mechanisms can be considered as separate entities, with minimal mutual-influence between the two mechanisms.

As the minimum effect of triboelectric layer surface area on the piezoelectric mechanism of the MT-HPTEH was noted, the effect on the voltage generated via triboelectric mechanism was evaluated. Table 2 shows the theoretical–experimental comparison of the output voltage by MT-HPTEH for various triboelectric surface areas studied. The percentage difference between the theoretical and experimental values of output voltage was in the range of 3 to 12%. The difference could be due to changes in triboelectric layer surface morphology as affected by repeated experiments^6,15,19^. Nevertheless, the theoretical–experimental deviations showed favourable outcome as the difference was less than 13% indicating accurate experimental data.

**Table 2:** Theoretical and experimental voltages from the triboelectric mechanism for different surface areas.

Area (cm^2^)	Effect of surface area		Percentage difference (%)
	Triboelectric open-circuit voltage (V)		
	Theoretical	Experimental	
3.0	1.316	1.479	12.38
4.0	2.641	2.542	3.75
9.0	4.381	4.134	5.64

**Figure 7:**
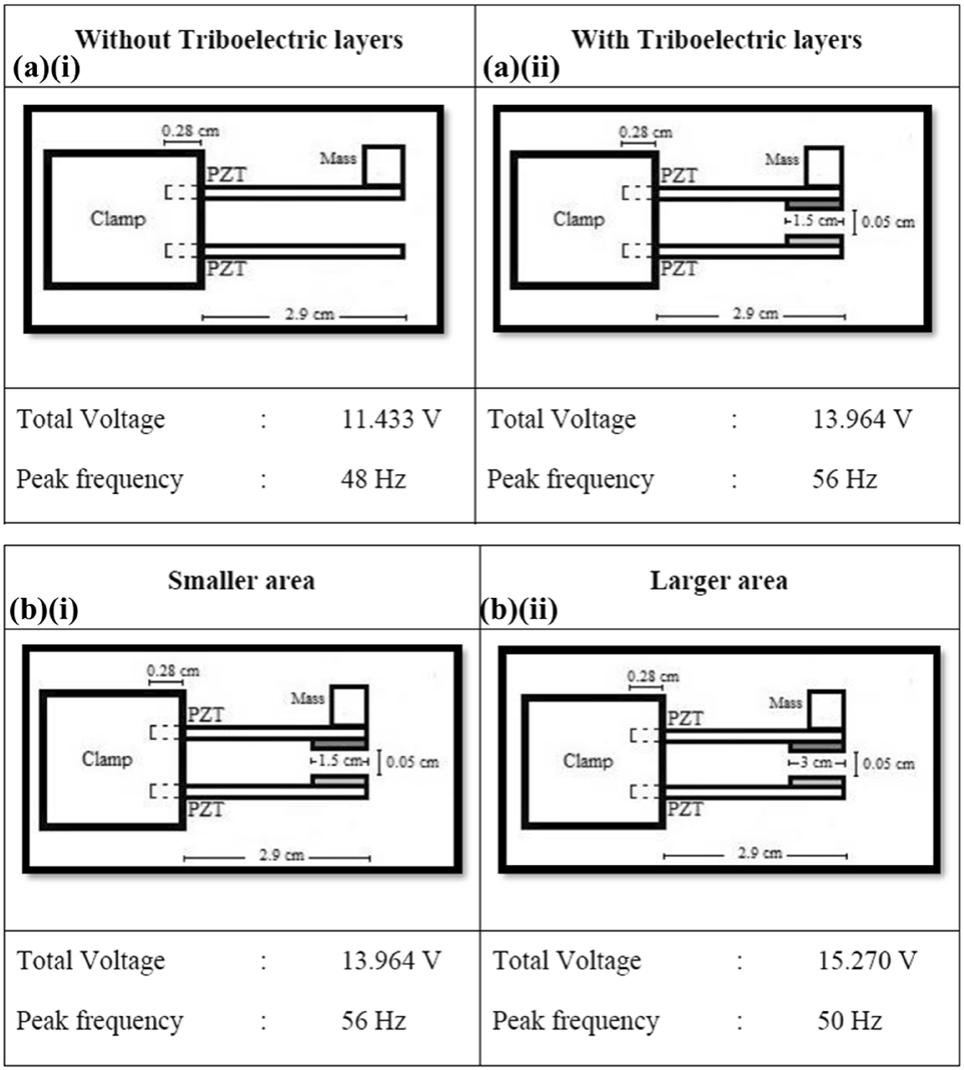
Performance enhancement of MT-HPTEH: (**a**) with and without triboelectric surface, (**b**) with different triboelectric area.

Comparison of total output voltages of MT-HPTEH as affected by various triboelectric layer conditions is shown in Fig. 7. From Fig. 7a, it can be observed that the output voltage of MT-HPTEH prototype increased significantly from 11.433 V to 13.964 V with the inclusion of triboelectric layers owing to the triboelectric energy harvesting mechanism as discussed in the previous sections and studies^3,13,34^. Meanwhile, Fig. 7b shows that the total output voltage improved from 13.964 V to 15.270 V as the Nylon and PTFE layer surface area increased from 3 cm^2^ to 9 cm^2^. These results suggest that additional electrical energy harvested via triboelectric mechanism contributes to the overall output voltage, consistent with the theoretical concepts outlined in Eqs. (3) to (6).

### Effect of extension length

**Figure 8:**
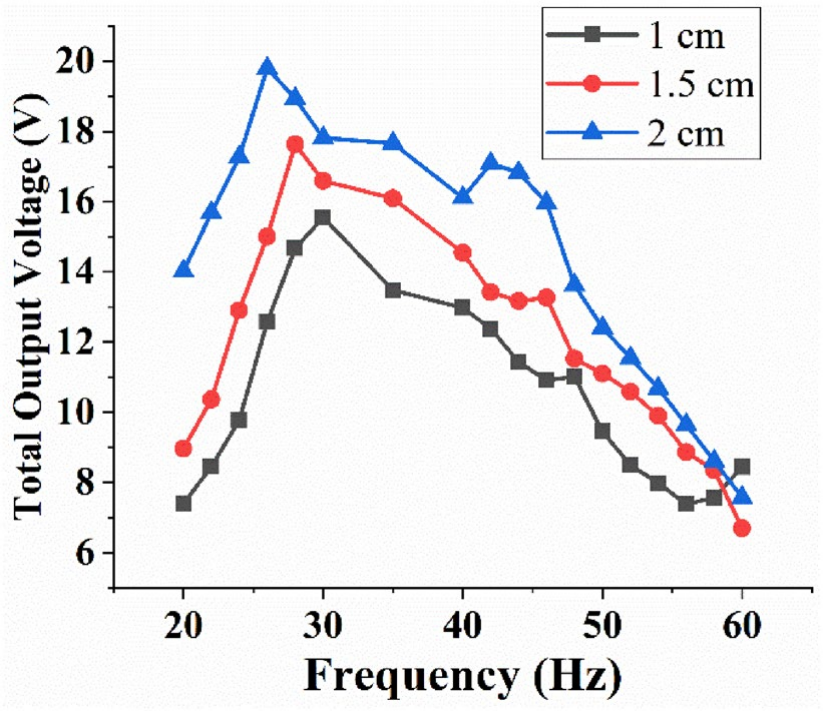
Effect of extension lengths on the total output voltage of MT-HPTEH.

As illustrated in Fig. 2, nylon extensions were attached to the piezoelectric beams in the MT-HPTEH to enhance its energy-harvesting performance based on the theory where the length of the beam can significantly affect the energy-harvesting performance of MT-HPTEH as related in Eqs. (1) and (3). It was predicted that the attachment of nylon extensions will increase the deflection and bonding stress in piezoelectric beams, resulting in higher output voltage. Besides, the deflection was expected to cause vigorous impact between triboelectric layers. In this study, extension lengths of 1, 1.5 and 2 cm were selected to determine the influence of extension lengths on the MT-HPTEH performance and the findings were exhibited in Fig. 8.

The total output voltage of MT-HPTEH was noted to increase with increasing extension lengths. As shown in Fig. 8, the highest peak voltage was obtained at ~ 20 V at a resonant frequency of 26 Hz for an extension length of 2 cm. The obtained voltage was higher than 1 cm and 1.5 cm counterparts, that harvested ~ 18 V at 28 Hz and ~ 16 V at 30 Hz, respectively. The increase in total output voltage with the usage of longer nylon extensions can be attributed to the occurrence of both piezoelectric and triboelectric mechanisms. For further understanding, the open-circuit voltage of the MT-HPTEH from the top piezoelectric beam, bottom piezoelectric beam, and triboelectric layers were also measured and benchmarked against the theoretical computation as shown in Table 3.

**Table 3:** Theoretical and experimental voltages from the top piezoelectric beam, bottom piezoelectric beam, and triboelectric mechanism for different extension lengths.

Extension(cm)	Top piezoelectric beam		Percentage difference (%)
	Theoretical	Experimental	
	Open-circuit voltage (V)	Open-circuit voltage (V)	
1.0	18.346	15.662	14.62
1.5	21.044	17.543	16.64
2.0	22.924	18.876	17.66

Extension(cm)	Bottom piezoelectric beam		Percentage difference (%)
	Theoretical	Experimental	
	Open-circuit voltage (V)	Open-circuit voltage (V)	
1.0	1.986	1.799	9.42
1.5	2.585	2.334	9.71
2.0	3.038	2.911	7.47

Extension(cm)	Triboelectric layer		Percentage difference (%)
	Theoretical	Experimental	
	Open-circuit voltage (V)	Open-circuit voltage (V)	
1.0	3.922	4.041	3.03
1.5	4.141	4.456	7.61
2.0	6.456	6.773	4.91

**Figure 9:**
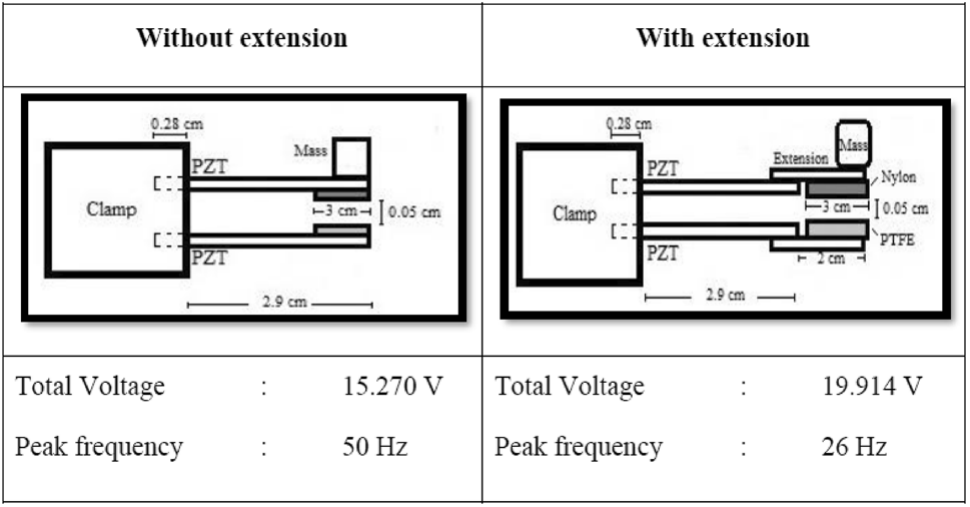
Performance enhancement of MT-HPTEH with nylon extension.

The open-circuit voltage from both piezoelectric and triboelectric mechanisms was found to increase significantly with higher extension length (Table 3). This confirmed the two hypotheses where longer nylon extensions will result in higher deflection and bonding stress in piezoelectric beams resulting in higher output voltage in the MT-HPTEH and the deflection will cause vigorous impact between triboelectric layers. The percentage difference between the theoretical and experimental output voltages ranged between 3 and 17%. The differences between the theoretical and experimental values could be due to external mechanism disruption which affects the energy-harvesting performances of the MT-HPTEH^18,35^. Nevertheless, the theoretical–experimental percentage differences were generally small (< 20%). This provides an engineering benchmark on the anticipated discrepancy when designing a MT-HPTEH from theoretical computation using Eqs. (1) and (3). Figure 9 shows the comparison of the total output voltages that shows the effect of extensions.

Integration of nylon extensions in MT-HPTEH to improve its energy-harvesting performance was further confirmed where the output voltage of MT-HPTEH prototype increased significantly from 15.270 V to 19.914 V with the inclusion of nylon extensions. At the same point, the peak frequency reduced from 50 to 26 Hz. This improvement can be attributed to the additional piezoelectric and triboelectric effects on the MT-HPTEH as displayed in Table 3.

### Effect of magnetic stiffness

Magnetic stiffness can be defined as the gap between two magnets integrated on the upper beam induced by repulsive magnetic forces as shown in Fig. 2b. In this study, the magnetic stiffness mainly focused on altering the natural frequency of the energy harvester for higher output. Equations (8) and (9) were used to establish the theoretical background to evaluate the effect of magnetic stiffness on the energy-harvesting performance of MT-HPTEH. The analysis was carried out with a tip mass integrated with a pair of magnets and placed vertically on both top and bottom piezoelectric beams as illustrated in Fig. 2.

**Figure 10:**
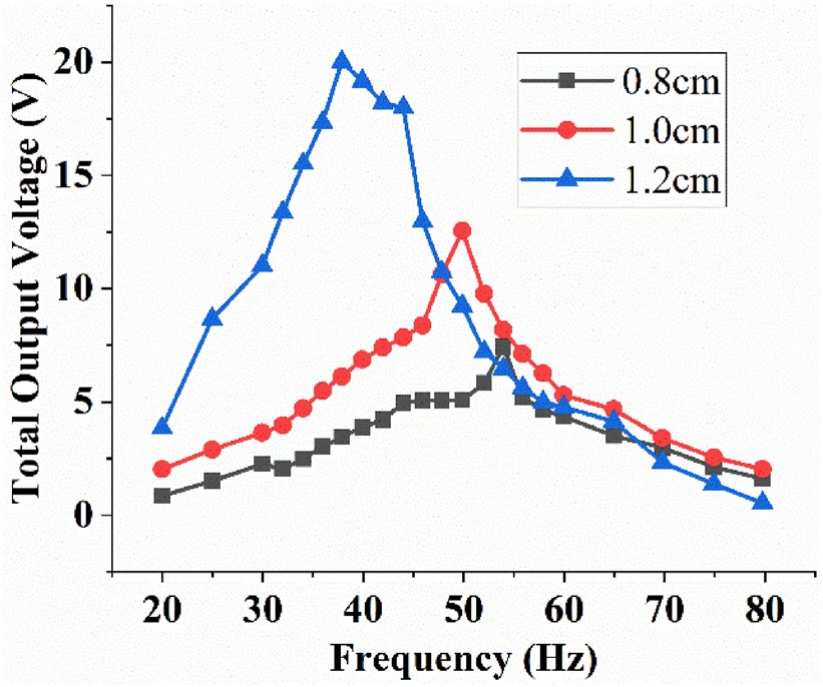
Effect of magnetic stiffness on the total output voltage of MT-HPTEH.

Figure 10 shows the effect of magnetic stiffness on the total output voltage of MT-HPTEH. The highest output voltage of 20 V was recorded at a 38 Hz of natural frequency when the magnetic stiffness was 1.2 cm. For magnetic stiffness of 0.8 cm and 1 cm, MT-HPTEH achieved output voltages of 13 V and 7 V at 48 Hz and 54 Hz of natural frequency, respectively. This result showed that the total output voltage of MT-HPTEH increased while the natural frequency decreased with increasing magnetic stiffness.

To investigate the effect of piezoelectric and triboelectric mechanisms on the magnetic stiffness and total output voltage variations, the open-circuit voltages from the top piezoelectric beam, bottom piezoelectric beam, and triboelectric layers were measured and compiled in Table 4 and for comparison purpose, theoretical values of open-circuit voltage were computed using Eqs. (8) and (9).

**Table 4:** Theoretical and experimental voltages from the top piezoelectric beam, bottom piezoelectric beam, and triboelectric mechanism under different magnetic stiffnesses.

Gap(cm)	Top piezoelectric beam		Percentage difference (%)
	Theoretical	Experimental	
	Open-circuit voltage (V)	Open-circuit voltage (V)	
0.8	6.08	6.317	12.55
1.0	11.058	10.023	9.36
1.2	15.555	17.174	10.41

Gap(cm)	Bottom piezoelectric beam		Percentage difference (%)
	Theoretical	Experimental	
	Open-circuit voltage (V)	Open-circuit voltage (V)	
0.8	1.986	1.659	16.46
1.0	2.585	2.187	11.99
1.2	3.038	2.869	5.56

Gap(cm)	Triboelectric layer		Percentage difference (%)
	Theoretical	Experimental	
	Open-circuit voltage (V)	Open-circuit voltage (V)	
0.8	2.025	1.775	12.35
1.0	3.163	3.354	6.04
1.2	6.152	6.754	9.78

The open-circuit voltages increased with increase in magnetic stiffness (Table 4). The voltage increment in the top piezoelectric beam was more prominent than the bottom beam as the magnetic tuning was exerted directly on the top beam. For the piezoelectric mechanism, magnetic stiffness of 1.2 cm resulted in lower repulsive which reduced the natural frequency, consistent with Eqs. (8) and (9). The reduction in natural frequency indirectly increased the amplitude of deflection in the piezoelectric beam for each contact hence improved the open-circuit voltage (Eq. 1). Meanwhile, for the triboelectric energy-harvesting mechanism, lower magnetic stiffness caused smaller distance between the triboelectric layers attached on upper and lower beams resulting in lower open-circuit voltage (Eq. 2).

The percentage difference between theoretical and experimental output voltages ranged between 3 to 16%. The differences could be due to magnetic flux disruption after repeated vibration which affected the energy-harvesting performance of the MT-HPTEH^22,33,36^. The theoretical–experimental discrepancy of MT-HPTEH (< 20%) serves as an important engineering benchmark when designing a MT-HPTEH.

**Figure 11:**
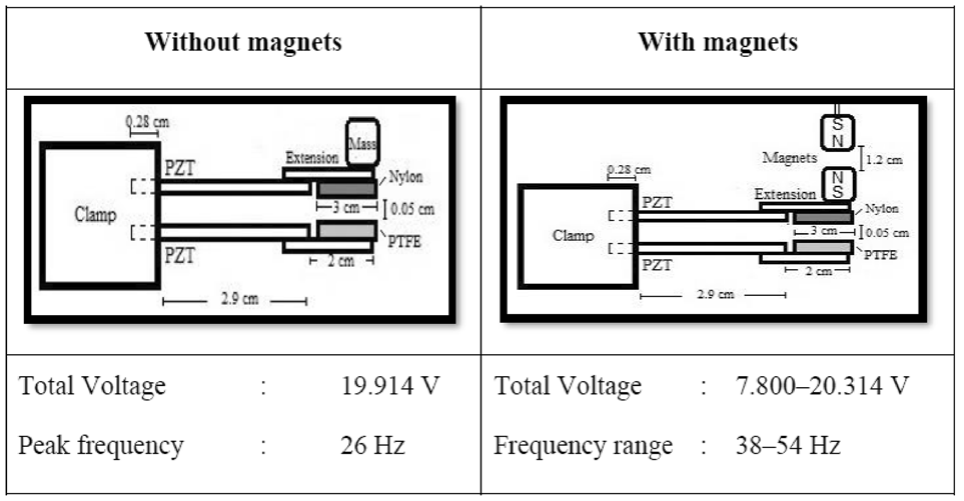
Performance enhancement of MT-HPTEH with magnet tuning.

Generally, magnetic tuning of piezoelectric energy harvester provides flexibility to adjust to the desired output voltage and natural frequency of MT-HPTEH. As shown in Fig. 11, the output voltage was tuned between 7.8 to 20 V while the natural frequency was tuned between 38 to 54 Hz. The flexibility of MT-HPTEH provides great insights into various applications, where specific working conditions, natural frequencies, and output voltages are required.

### Overall performance of optimised MT-HPTEH

**Figure 12:**
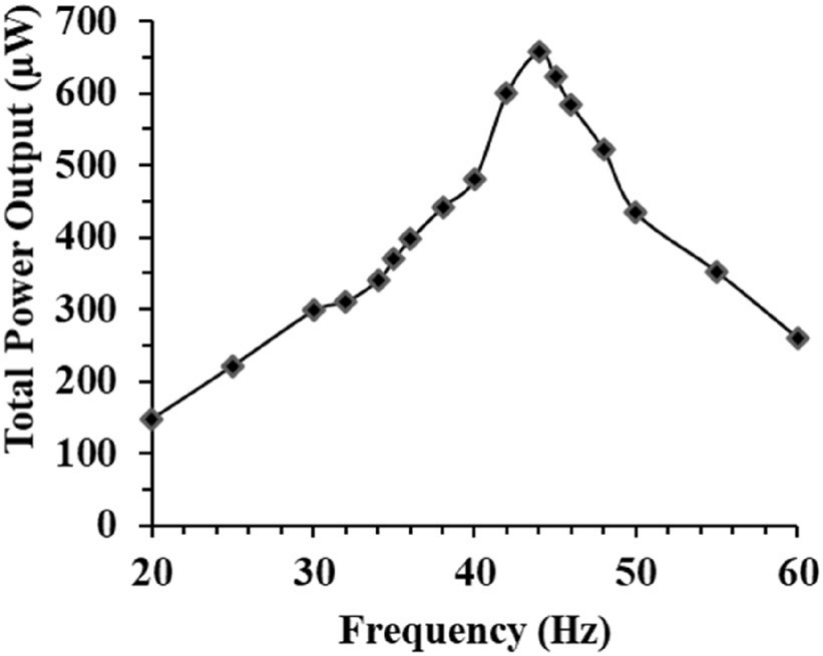
Total power output of MT-HPTEH against frequency load of 180 kΩ.

**Figure 13:**
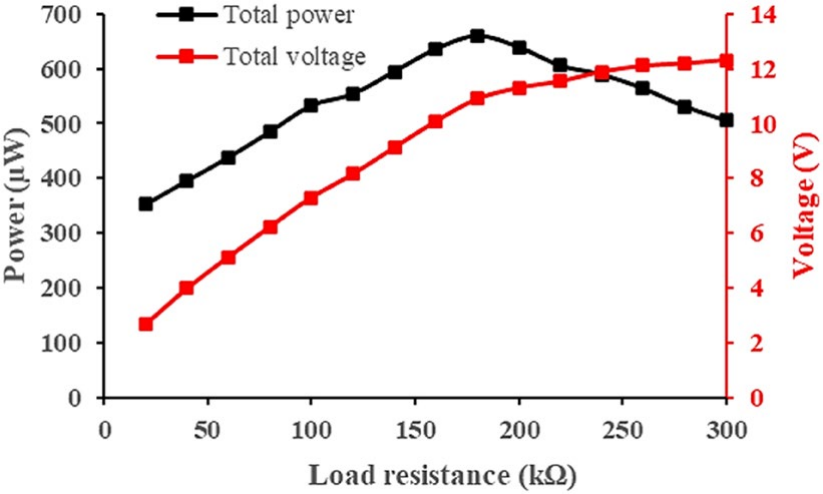
Total power and voltage output of MT-HPTEH against load resistances.

Based on the abovementioned key design factors such as mass placement, triboelectric surface area, extension length, and magnetic stiffness, overall power generated by the optimised MT-HPTEH is shown in Fig. 12. Under 180 kΩ load, the optimised MT-HPTEH achieved a peak output power of ~ 659 µW at a peak frequency of ~ 36 Hz. Figure 13 shows the power and voltage produced at various load resistances.

Theoretical and experimental powers from the top piezoelectric beam, bottom piezoelectric beam, and triboelectric mechanism of optimised MT-HPTEH are shown in Table 5. The highest power was generated from the top piezoelectric beam as the effect of prominent key design factors (magnetic stiffness and mass placement) were more significant on the top piezoelectric beam. The percentage difference between theoretical and experimental power values was relatively low (< 10%) indicating the developed MT-HPTEH can be optimised via design factors such as mass placement, triboelectric layer surface area, extension length and magnetic stiffness. The theoretical–experimental deviation also provides an engineering benchmark on the possible performance errors in designing MT-HPTEH which could suggest for further design and process optimisation for improvements.

**Table 5:** Theoretical and experimental power from the top piezoelectric beam, bottom piezoelectric beam, and triboelectric mechanism of optimised MT-HPTEH.

Mechanism	Theoretical power (μW)	Experimental power (μW)	Percentage difference (%)
Top piezoelectric	340	360	5.88
Bottom piezoelectric	5.29	4.8	9.26
Triboelectric	3.36	3.51	4.46

## Conclusion

Magnetically-tunable hybrid piezoelectric-triboelectric energy harvester (MT-HPTEH) was designed and optimised in this study. The optimisation was carried out based on four key design factors which were mass placement, triboelectric layer surface area, extension length and magnetic stiffness. The energy harvesting performance of MT-HPTEH was based on piezoelectric and triboelectric mechanisms. The higher extension length and magnetic stiffness resulted in a higher output voltage of both piezoelectric and triboelectric entities. Besides, higher mass on MT-HPTEH beams significantly increased the open voltage from the piezoelectric mechanism, while higher triboelectric surface area significantly increased the open voltage from the triboelectric mechanism. The theoretical–experimental voltage differences were < 20%, whereas < 10% discrepancy of theoretical–experimental power generated by optimised MT-HPTEH was noted. The findings from this study provide a comprehensive insight into the mechanical design of MT-HPTEH which can serve as a preliminary guideline for the future design of piezo-tribo integrated energy harvesters.
